# Supplementary material for: Limits to reproduction and seed size-number trade-offs that shape forest dominance and future recovery
Source: Nat Commun. 2022 May 2;13:2381. doi: 10.1038/s41467-022-30037-9 (PMC9061860; doi:10.1038/s41467-022-30037-9)
Supplement: Supplementary file 1 — Supplementary information [file 41467_2022_30037_MOESM1_ESM.pdf]

Supplementary information

Tong Qiu, Robert Andrus, Marie-Claire Aravena, Davide Ascoli, Yves Bergeron, Roberta Berretti, Daniel Berveiller, Michal Bogdziewicz, Thomas Boivin, Raul Bonal, Don C. Bragg, Thomas Caignard, Rafael Calama, J. Julio Camarero, Chia-Hao Chang-Yang, Natalie L. Cleavitt, Benoit Courbaud, Francois Courbet, Thomas Curt, Adrian J. Das, Evangelia Daskalakou, Hendrik Davi, Nicolas Delpierre , Sylvain Delzon, Michael Dietze, Sergio Donoso Calderon, Laurent Dormont, Josep Espelta, Timothy J. Fahey, William Farfan-Rios, Catherine A. Gehring, Gregory S. Gilbert, Georg Gratzer, Cathryn H. Greenberg, Qinfeng Guo, Andrew Hacket-Pain, Arndt Hampe , Qingmin Han, Janneke Hille Ris Lambers, Kazuhiko Hoshizaki, Ines Ibanez, Jill F. Johnstone, Valentin Journe, Daisuke Kabeya, Christopher L. Kilner, Thomas Kitzberger, Johannes M.H. Knops, Richard K. Kobe, Georges Kunstler, Jonathan G.A. Lageard, Jalene M. LaMontagne, Mateusz Ledwon, Francois Lefevre , Theodor Leininger, Jean-Marc Limousin, James A. Lutz, Diana Macias, Eliot J.B. McIntire, Christopher M. Moore, Emily Moran, Renzo Motta, Jonathan A. Myers, Thomas A. Nagel, Kyotaro Noguchi, Jean-Marc Ourcival , Robert Parmenter, Ian S. Pearse, Ignacio M. Perez-Ramos, Lukasz Piechnik, John Poulsen, Renata Poulton-Kamakura, Miranda D. Redmond, Chantal D. Reid, Kyle C. Rodman, Francisco Rodriguez-Sanchez, Javier D. Sanguinetti , C. Lane Scher, William H. Schlesinger, Harald Schmidt Van Marle, Barbara Seget, Shubhi Sharma, Miles Silman, Michael A. Steele, Nathan L. Stephenson, Jacob N. Straub, I-Fang Sun, Samantha Sutton, Jennifer J. Swenson, Margaret Swift, Peter A. Thomas, Maria Uriarte, Giorgio Vacchiano, Thomas T. Veblen, Amy V. Whipple, Thomas G. Whitham, Andreas P. Wion, Boyd Wright, S. Joseph Wright, Kai Zhu, Jess K. Zimmerman, Roman Zlotin, Magdalena Zywiec, James S. Clark

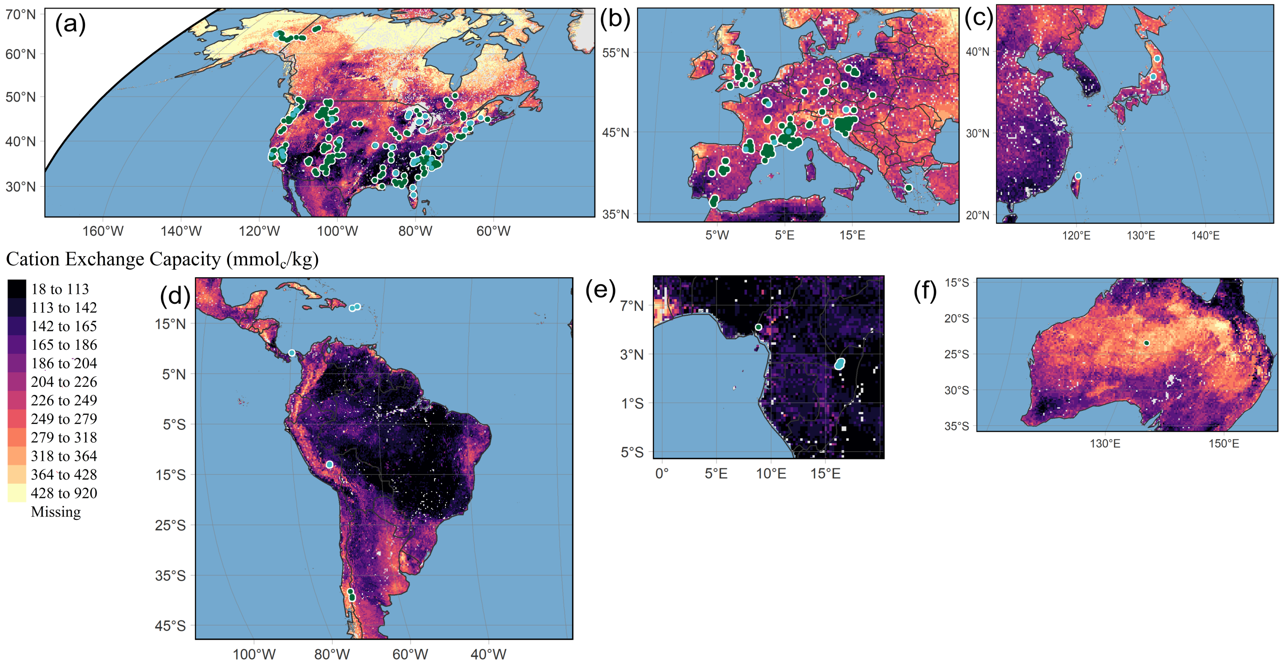

Supplementary Figure 1: Global cation exchange capacity map (at soil depth of 0 to 30cm) and the MASTIF sites in (a) North America, (b) Europe, (c) Asia, (d) Central and South America, (e) Africa, and (f) Oceania. Blue and green points indicate sites that collected seed trap (ST) and crop count (CC) data, respectively.

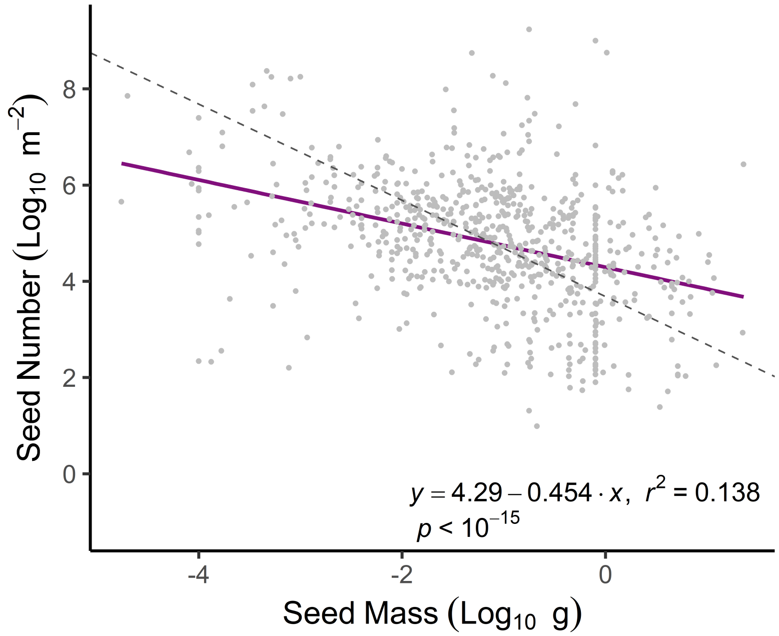

Supplementary Figure 2: A negative trend between seed number (seeds per m^2 tree basal area, log_{10} scale) and seed mass (g, log_{10} scale) across 714 tree species does not approach a strict trade-off (dashed line with slope -1). The standard errors for the coefficients (intercept and slope) are 0.065 and 0.042, respectively. The p-value is obtained from a two-tailed test.

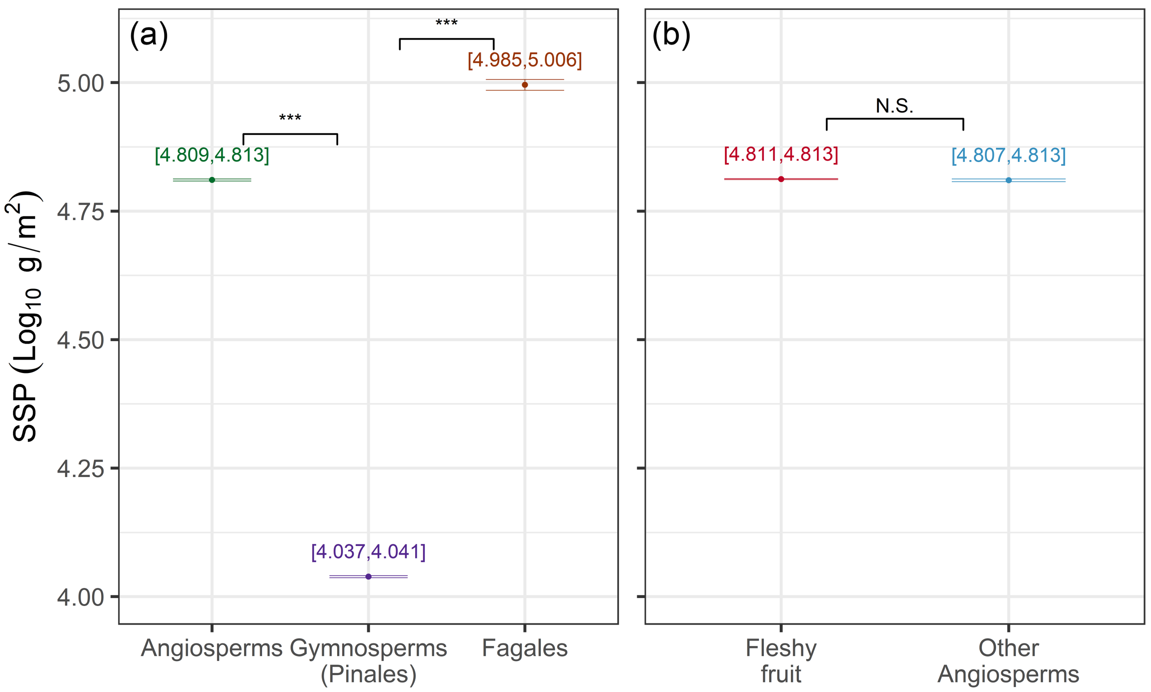

Supplementary Figure 3: Comparison of group seed production (GSP, g seed per m^2 tree basal area, see methods) of Fig. 2b (a) between angiosperms ($n = 135,616$, gymnosperms (i.e., Pinales, $n = 32,559$), and Fagales ($n = 13,016$); and (b) between fleshy fruits ($n = 33,815$) and other angiosperms ($n = 101,801$). Point shows weighted mean of GSP and two lines indicate the standard error. Texts are used to label the mean $\pm$ standard error because standard errors are small. The Welch's two-sample T-test indicates that angiosperms have a significantly higher mean GSP than gymnosperms ($p < 10^{-16}$). Large seeded Fagales also had higher mean GSP than the cone-producing Pinales ($p < 10^{-16}$). There are no significant differences between fleshy fruit species and other angiosperms ($p = 0.44$). SSP in the y axis is species seed production from main text.

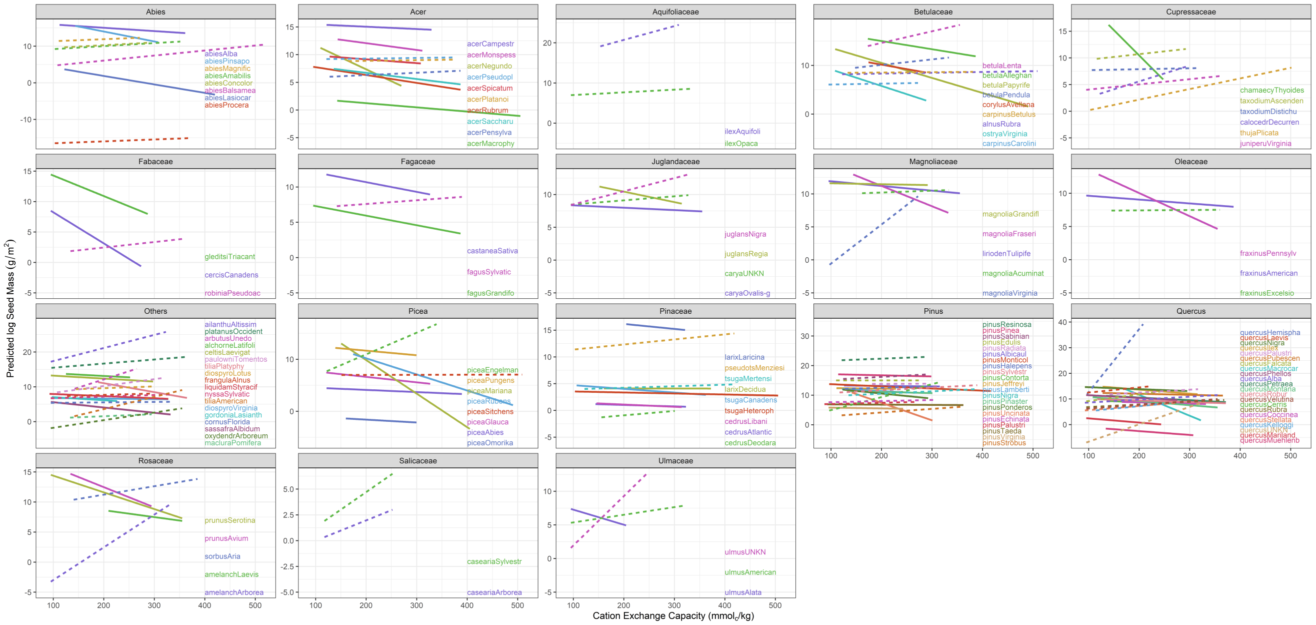

Supplementary Figure 4: Within-species response to cation exchange capacity (β_{cec}) and the range of soil fertility for tree species observed in the Masting Inference and Forecasting (MASTIF) network. Each line is a prediction along the soil fertility gradient from the fitted model by holding constant values for other covariates. Specifically, diameter is set to half of the maximum diameter observed for the species; canopy class is set to the intermediate shade class (3 on a 1-5 scale); temperature and moisture deficit are set to mean observed values for the species. Species are grouped by genus or family, distinguished by color. Solid lines indicate reproductive investment declines with increasing CEC (negative β_{cec}). Dashed lines represents positive β_{cec}.

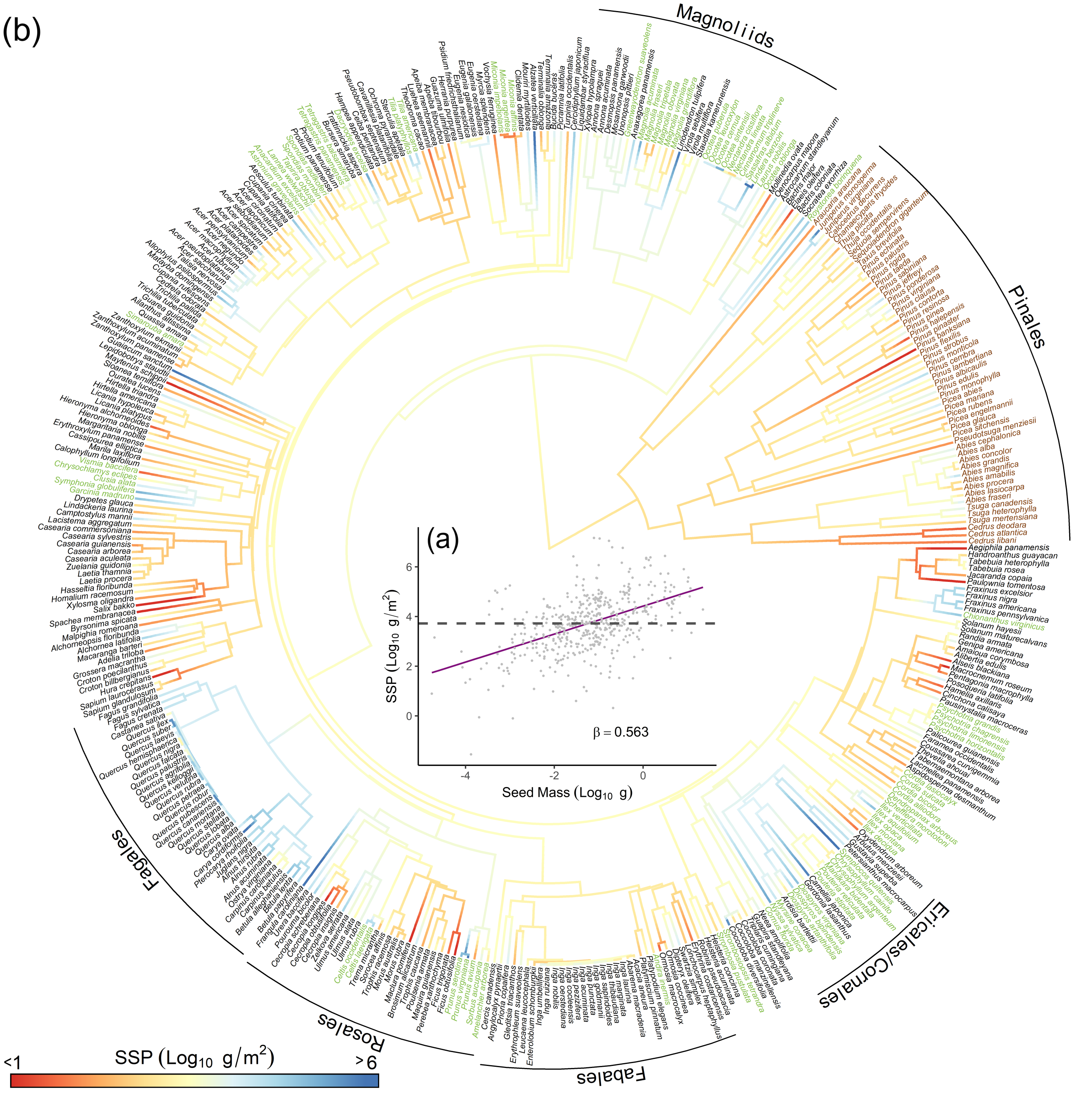

Supplementary Figure 5: After removing species that used genus- and family-level mean of seed mass, conclusions still remain consistent with the main texts. Specifically, (a) Species seed production (SSP, g seed per m^2 tree basal area) has a positive correlation with seed mass across 714 tree species ($\log_{10}\mathrm{SSP} = 4.42 + 0.563 \cdot \log_{10} m$, $R^2 = 0.246$, $p < 10^{-15}$, $n = 480$). (b) SSP exhibits strong phylogenetic coherence for 391 species where we have phylogeny data (81% of 480 species). The phylogenetic signal estimated using $Pagel$'s $\lambda = 0.73$ ($p < 10^{-10}, n = 391$).

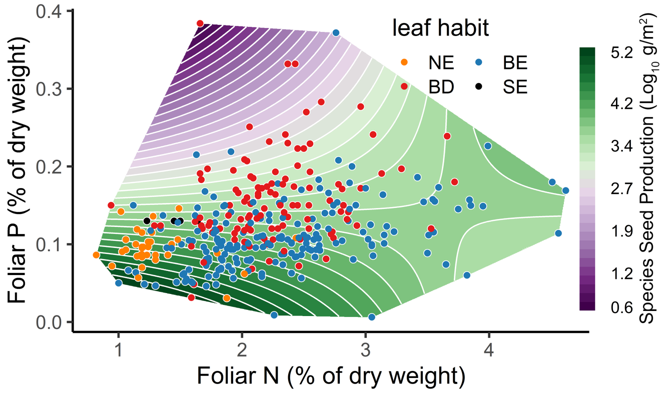

Supplementary Figure 6: After removing species that used genus- and family-level mean of seed mass, effects of foliar nitrogen (N) and phosphorus (P) on SSP (g seed per m^2 tree basal area) still remains consistent with the main texts. SSP decreases approximately four orders of magnitude along the foliar P gradient while effects of foliar N remain discernable.

Supplementary tables

Supplementary Table 1: Species seed production (SSP, g seed per m^2 tree basal area) related to foliar nitrogen (N), foliar phosphorus (P), and leaf habit in a phylogenetic regression (adjusted $R^2 = 0.22$ for 427 species where we have both foliar nutrients and phylogeny data). Needleleaf evergreen is the reference level for the leaf-habit factor. Modeling of SSP is on the proportionate scale (log). The p-value is calculated from a two-tailed test.

	Estimate	Std	t-value	p-value
Intercept	11.9	11.1	1.07	0.28
Foliar N	-1.07	0.492	-2.17	0.031
Foliar P	-34.8	9.13	-3.81	$<$0.001
Foliar N $\times$ P	8.09	3.31	2.45	0.015
Broadleaf deciduous	1.20	1.69	0.711	0.48
Broadleaf evergreen	0.0278	1.69	0.0164	0.99
Scalelike evergreen	1.76	13.5	0.131	0.90

Supplementary Table 2: Sites with tree-years listed by World Wildlife Fund (WWF) ecoRegion. The 'Data' column indicates seed traps (ST) and crop counts (CC). Ecoregion names are followed by numbers of opportunistic observations that are not on longitudinal plots.

Eco-region (opportunistic)	Site	PI(s)	lon	lat	tree-Yr	Data	Citation
**A/W Turkey sclerophyllous/mixed (97)**							
	PNPG	E. Daskalakou	24	38	899	CC	[9]
**Alps conifer/mixed forests** (724)							
.	ROTH	G. Gratzer	15	48	34752	ST	
	BELLE	G. Kunstler; B. Courbaud	6	45	13590	ST CC	
	RNNPT	L. Dormon	7	45	434	CC	[13]
	PNPP	D. Ascoli; R. Motta; R. Berretta; G. Vacchiano	12	46	7391	ST	[15]
**Appalachian-Blue Ridge forests** (216)							
	BCEF	C. Greenberg	-83	35	5486	CC	[28]
	CWT	J. S. Clark	-83	35	309825	ST CC	[6]
	EPENN	M. Steele	-76	41	2191	CC	
	GRAN	C. Greenberg	-82	36	623	CC	[28]
	GRSM	J. S. Clark	-83	36	27474	ST	
	MARS	J. S. Clark; Scott Pearson	-83	36	33512	ST CC	[7]
	MLBS	J. S. Clark	-81	37	967	ST CC	
	PISG	C. Greenberg	-83	35	1890	CC	[28]
	SCBI	B. McShea	-78	39	74481	ST	[3]
**Appalachian mixed mesophytic forests** (53)							
**Arizona Mountains forests** (9)							
	MOPA	M. Redmond	-106	33	110	CC	[26]
	REMO	A. Whipple; C. Gering; T. Whitham	-112	36	1050	CC	[34]
	SICI	A. Wion; M. Redmond	-108	33	168	CC	
	WHIT	A. Wion; M. Redmond	-109	33	280	CC	
	WINO	A. Whipple; C. Gering; T. Whitham	-111	35	669	CC	[34]
**Atlantic coastal pine barrens** (29)							
	BARBEAU	N. Delpierre; D. Berveiller	3	48	2121	ST	
**Atlantic mixed forests** (806)							
**Balkan mixed forests** (25)							
**Baltic mixed forests** (16)							
**British Columbia mainland coastal forests** (73)							
	GLCR1	J. Franklin	-122	49	1607	CC	
	GLCR2	J. Franklin	-122	49	1391	CC	
	HEME	J. Franklin	-122	49	1273	CC	
	STPA	J. Franklin	-121	47	4195	CC	
**California Central Valley grasslands** (19)							
**California interior chaparral/woodlands** (285)							

Continued on next page

Table Supplementary Table 2 – continued from previous page

Eco-region (opportunistic)	Site	PI(s)	lon	lat	tree-Yr	Data	Citation
**California montane chaparral/woodlands** (60)							
	HNHR	J. Knops; W. Koenig	-122	36	200	CC	[20]
**Carpathian montane forests**							
	BGNP	M. Zywiec; L. Piechnik; B. Seget; M. Ledwon	20	50	5619	CC	
**Cascade Mountains leeward forests**							
	TUCR	J. Franklin	-121	48	2587	CC	
**Celtic broadleaf forests**							
	BENWE	A. HacketPain	-2	55	275	CC	[2]
	CONGL	A. HacketPain	-2	53	16	CC	[2]
	GILLF	A. HacketPain	-2	54	24	CC	[2]
	HIMLE	A. HacketPain	-2	53	155	CC	[2]
	KEELE	A. HacketPain	-2	53	20	CC	[2]
	KILLE	A. HacketPain	-3	51	171	CC	[2]
	RIPON	A. HacketPain	-1	54	347	CC	[2]
	SPENN	A. HacketPain	-2	55	289	CC	[2]
	WOODB	A. HacketPain	-3	51	262	CC	[2]
**Central Canadian Shield forests**							
	COCH	Y. Bergeron; Y. Messaoud	-81	49	22	CC	[21]
	LDUPT	Y. Bergeron; Y. Messaoud	-79	48	20	CC	[21]
	MASK	Y. Bergeron; Y. Messaoud	-79	50	22	CC	[21]
**Central European mixed forests** (158)							
**Central forest-grasslands transition** (284)							
	UKFS	J. S. Clark	-95	39	218	ST CC	
	WUSL	J. Myers	-91	39	115937	ST	
**Central Pacific coastal forests** (63)							
	MAPK	J. Franklin	-124	45	1643	CC	
**Central Ranges xeric scrub**							
	LS	Boyd Wright	132	-24	36	CC	[36]
**Central tall grasslands** (24)							
**Central/S Cascades forests** (41)							
	BAMT	J. Franklin	-122	46	1517	ST	
	BERK	J. Franklin	-122	43	3456	CC	
	BLLK	J. Franklin	-122	46	1265	CC	
	DECU	J. Franklin	-122	45	1095	CC	
	IRMT	J. Franklin	-122	44	1223	CC	
	MOLK	J. Franklin	-122	46	1411	CC	
	MORA	J. HilleRisLambers	-122	47	63395	ST	
	PEPR	J. Franklin	-122	46	1813	CC	

Continued on next page

Table Supplementary Table 2 – continued from previous page

Eco-region (opportunistic)	Site	PI(s)	lon	lat	tree-Yr	Data	Citation
	SAMT	J. Franklin	-122	44	1315	CC	[26]
	SAPA	J. Franklin	-122	45	2445	CC	
	SIRK	J. Franklin	-122	46	969	CC	
	SLBE	J. Franklin	-122	46	1252	CC	
	STMT	J. Franklin	-122	46	2479	CC	
	TIRD	J. Franklin	-122	46	1071	CC	
	WIMT1	J. Franklin	-122	44	1153	CC	
	WIMT2	J. Franklin	-122	44	1532	CC	
	WISP	J. Franklin	-122	43	1331	CC	
	WREF	J. S. Clark; J. HilleRisLambers	-122	46	83706	ST CC	
**Chihuahuan desert**							
	FOBA	M. Redmond	-108	33	150	CC	[26]
**Colorado Plateau shrublands** (1)							
	ALBU	A. Wion; M. Redmond	-106	35	168	CC	[35]
	CEBO	A. Wion; M. Redmond	-106	36	182	CC	[27]
	DOLO	A. Wion; M. Redmond	-109	38	210	CC	[27]
	GLPA	A. Wion; M. Redmond	-109	39	210	CC	[27]
	HOND	A. Wion; M. Redmond; K. Rodman	-106	37	416	CC	
	HOTC	A. Wion; M. Redmond; K. Rodman	-108	39	196	CC	[27]
	LASA	A. Wion; M. Redmond	-109	39	210	CC	[27]
	MAGD	A. Wion; M. Redmond	-107	34	294	CC	[27]
	MONT	A. Wion; M. Redmond	-108	38	182	CC	[27]
	NATU	A. Wion; M. Redmond	-109	38	182	CC	[27]
	SEV	R. Zlotin; Diana Macias	-107	34	5995	CC	[23]
	SUCR	A. Whipple; C. Gering; T. Whitham	-111	36	6152	CC	[34]
**Colorado Rockies forests** (135)							
	BOCA	Ian Pearse	-105	40	6919	CC	
	CANJ	A. Wion; M. Redmond	-106	36	192	CC	[27]
	HAYM	A. Wion; M. Redmond	-105	39	448	CC	
	LAK	K. Rodman	-106	36	255	CC	[27]
	LV	M. Redmond	-105	36	110	CC	[26]
	MG	K. Rodman	-106	36	204	CC	[27]
	MON	K. Rodman	-106	36	289	CC	[27]
	MR	K. Rodman	-106	36	255	CC	[27]
	MVG	K. Rodman	-106	36	255	CC	[27]
	NIWO	J. S. Clark	-106	40	15321	ST CC	
	PC	K. Rodman	-106	36	289	CC	[27]
	PECO	M. Redmond	-106	36	120	CC	[26]

Continued on next page

Table Supplementary Table 2 – continued from previous page

Eco-region (opportunistic)	Site	PI(s)	lon	lat	tree-Yr	Data	Citation
	POND	A. Wion; M. Redmond	-107	36	168	CC	[35]
	RATN	M. Redmond	-104	37	150	CC	[26]
	SAFE	A. Wion; M. Redmond	-106	36	282	CC	[27]
	WACA	A. Wion; M. Redmond	-105	39	80	CC	
	WEMO	A. Wion; M. Redmond	-105	38	224	CC	
**Cross-Sanaga-Bioko coastal forests**							
	KNP	J. Norghauer	9	5	234	CC	[22]
**Dinaric Mountains mixed forests** (230)							
**E Cascades forests** (15)							
**E forest-boreal transition** (49)							
	LMONT	Y. Bergeron; Y. Messaoud	-79	48	23	CC	[21]
**E Great Lakes lowland forests** (17)							
**English Lowlands beech forests**							
	BEECH	A. HacketPain	0	52	60	CC	[2]
	BUCKH	A. HacketPain	-2	52	291	CC	[2]
	FISHH	A. HacketPain	-2	52	140	CC	[2]
	NETTL	A. HacketPain	-1	52	236	CC	[2]
	PAINS	A. HacketPain	-2	52	137	CC	[2]
	PATCH	A. HacketPain	-0	51	230	CC	[2]
	STP	M. Fenner M. Hanley	-1	51	296	CC	[18]
**Great Basin shrub steppe**							
	DSP	M. Redmond	-119	39	384	CC	
**Iberian sclerophyllous/semi-deciduous** (67)							
	CARB	C. Perez-Izquierdo	-6	40	402	CC	
	HUEC	R. Bonal	-4	40	183	CC	
	SIOE	R. Calama	-4	40	2672	CC	
	VALT	J. Espelta	-4	41	5400	CC	
**Illyrian deciduous forests** (62)							
**Interior Alaska-Yukon lowland taiga**							
	EAPL	J. Johnstone	-137	66	60	CC	[33]
	BONA	J. Johnstone	-148	65	34861	ST	
	FAIR	J. Johnstone	-148	65	159	CC	[33]
	LAGE	J. Johnstone	-145	64	51	CC	[33]
	LELA	J. Johnstone	-138	64	54	CC	[33]
	SMR	J. Johnstone	-141	64	18	CC	[33]
**Interior Yukon-Alaska alpine tundra**							
	CHIC	J. Johnstone	-143	63	48	CC	[33]
**Isthmian-Atlantic moist forests**							

Continued on next page

Table Supplementary Table 2 – continued from previous page

Eco-region (opportunistic)	Site	PI(s)	lon	lat	tree-Yr	Data	Citation
	BCI	S.J. Wright	-80	9	7947382	ST	
**Italian sclerophyllous/semi-deciduous** (159)							
**Klamath-Siskiyou forests**							
	ASRN	J. Franklin	-123	42	1635	CC	
	MEOV	J. Franklin	-123	42	1631	CC	
**Mid Atlantic coastal forests** (316)							
	BLSF	D. Brockway	-79	35	354	CC	[5]
	CALL	J. S. Clark	-79	35	293	ST CC	
	CROA	S. Cohen	-77	35	280	CC	
	GRSW	J. S. Clark	-78	34	38	ST	
	SASF	D. Brockway	-81	34	314	CC	[5]
**Mississippi lowland forests**							
	CHICK	J. Straub; T. Leininger	-90	36	133	CC	[30]
	DELTA	J. Straub; T. Leininger	-91	33	260	CC	[30]
	MINGO	J. Straub; T. Leininger	-90	37	146	CC	[30]
	TENAS	J. Straub; T. Leininger	-91	32	106	CC	[30]
	WHITE	J. Straub; T. Leininger	-91	34	50	CC	[30]
**Montana Valley/Foothill grasslands** (1)							
**N California coastal forests** (6)							
	UCSC	G. Gilbert; Kai Zhu	-122	37	213228	ST	
**N Central Rockies forests**							
	WBP	E. McIntire	-114	48	5225	CC	
**N short grasslands** (4)							
**NE coastal forests** (58)							
**NE Spain/S France Mediterranean** (454)							
	ISS	H. Davi	6	44	676	CC	[10]
	PCMEJEAN	T. Curt	3	44	76	CC	[12]
	PUECHEXP1	J. Limousin; J. Ourcival	4	44	3300	ST	
	RBI	T. Boivin	5	44	540	CC	[14]
	RBLL	H. Davi	6	44	250	CC	[10]
	VALLI	F. Lefevre; F. Courbet	5	44	1900	CC	
	VEN	H. Davi	5	44	4899	CC	[10]
	VENT	H. Davi	5	44	192	CC	[10]
	VES	H. Davi	7	44	491	CC	[10]
**New England-Acadian forests** (161)							
	ASWP	C. Moore; J. S. Clark	-69	45	626	ST CC	
	BART	I. Fer; M. Dietze	-71	44	26800	ST	
	COMPT	W. Schlesinger	-67	45	59	CC	

Continued on next page

Table Supplementary Table 2 – continued from previous page

Eco-region (opportunistic)	Site	PI(s)	lon	lat	tree-Yr	Data	Citation
	HARV	J. S. Clark	-72	42	21671	ST CC	
	HBEF	T. Fahey; N. Cleavitt	-72	44	22393	ST	[8]
**Nihonkai montane deciduous forests**							
	KANU	K. Hoshizaki	141	39	43395	ST	
	JNP	Q. Han; D. Kabeya; K. Noguchi	139	37	602	ST	[17]
**NW Congolian lowland forests**							
	CONGO	J. Poulson; C. Nunez	16	2	13785	ST	
**Pannonian mixed forests** (431)							
**Peruvian Yungas**							
	ABERG	M. Silman; W. Farfan	-72	-13	45459	ST	
**Piney Woods forests**							
	KINF	D. Brockway	-92	31	291	CC	[5]
**Pontic steppe** (42)							
**Puerto Rican dry forests**							
	GUA	M. Uriarte	-67	18	162700	ST	[31]
**Puerto Rican moist forests**							
	LUQ	M. Uriarte	-66	18	169125	ST	[31]
**Puget lowland forests** (61)							
**Pyrenees conifer/mixed forests** (28)							
	PNVO	J. Camarero	-1	43	1458	ST	[11]
	PNP	S. Delzon; T. Caignard	-0	43	205	CC	[4]
**Rodope montane mixed forests** (7)							
**S Central Rockies forests** (136)							
	YELL	J. S. Clark	-110	45	439	ST CC	
**S Great Lakes forests**							
	ANNA	I. Ibanez	-84	42	36235	ST	[26]
**SE conifer forests** (143)							
	APNF	D. Brockway	-85	30	249	CC	[5]
	BRSF	D. Brockway	-87	31	148	CC	[5]
	DSNY	J. S. Clark	-81	28	115	ST CC	
	EAFB	D. Brockway	-87	30	890	CC	[5]
	EEF	D. Brockway	-87	31	1086	CC	[5]
	JERC	D. Brockway	-84	31	176	CC	[5]
	OSBS	J. S. Clark	-82	30	844	ST CC	
	STCB	D. Brockway	-85	31	35	CC	[5]
	TTRS	D. Brockway	-86	31	92	CC	[5]
**SE mixed forests** (1591)							
	DUKE	J. S. Clark	-79	36	634247	ST CC	[1]

Continued on next page

Table Supplementary Table 2 – continued from previous page

Eco-region (opportunistic)	Site	PI(s)	lon	lat	tree-Yr	Data	Citation
	FBMB	D. Brockway	-85	32	650	CC	[5]
	SERC	J. S. Clark	-77	39	555	ST CC	[32]
	TALL	J. S. Clark	-87	33	484	ST CC	
**Sierra Nevada forests** (381)							
	SEQU	A. Das; N. Stephenson	-119	37	229934	ST	[32, 37, 38]
	SOAP	J. S. Clark	-119	37	647	ST CC	
	YOSE	A. Das; N. Stephenson	-120	38	90807	ST	[32, 37, 38]
**SW Iberian Mediterranean S/M** (152)							
	ALCO	I. Perez-Ramos	-6	36	446	CC	[24]
	PNLA	A. Hampe	-6	37	1006	CC	[16]
	SJDV	F. Rodriguez-Sanchez	-6	37	624	CC	
**Taiwan subtropical evergreen forests**							
	FFDF	C. Chang-Yang; I-Fang Sun	122	25	1338605	ST	
**Upper Midwest forest-savanna transition** (5)							
**Valdivian temperate forests**							
	LNP	J. Sanguinetti; T. Kitzberger	-71	-39	76	CC	[29]
	ARAU	M. Aavena; S. DonosoCalderon	-71	-38	2070	CC	
**W European broadleaf forests** (1591)							
**W Great Lakes forests** (65)							
	CADI	R. Kobe	-86	44	8388	ST CC	
	MANI	R. Kobe	-86	44	9840	ST CC	
	MICH	J. LaMontagne	-88	47	1008	CC	
	PAFA	J. LaMontagne	-90	46	7	CC	
	TREE	J. S. Clark	-90	45	542	ST CC	
	UMBS	I. Ibanez	-85	46	68360	ST	
	UNDE	J. S. Clark	-90	46	18667	ST CC	
	WILW	J. LaMontagne	-90	46	7	CC	
	WORU	J. LaMontagne	-90	46	623	CC	
**W Gulf coastal grasslands** (26)							
**W short grasslands** (1)							
	CMNM	M. Redmond	-104	37	120	CC	[26]
	KENT	M. Redmond	-103	37	210	CC	[26]
**Willamette Valley forests**							
	CAMT	J. Franklin	-123	45	1226	CC	
**Wyoming Basin shrub steppe**							
	NORT	A. Wion; M. Redmond	-109	41	182	CC	

Supplementary Table 3: Covariates in the MASTIF model, not all of which are important for all species. Individual attributes include diameter D and shade S. Climate variables includes temperature T and moisture deficit M. Habitat conditions include cation exchange capacity CEC and local drainage $u1$, $u2$, and $u3$ (see methods). Subscripts are tree i, month m, stand j, and year t. PET and P represents potential evapotranspiration and precipitation, respectively.

Predictors	Symbol	Dimensions	Definitions	Source
Diameter	$D_{ij,t}$	cm		MASTIF
	$D^2_{ij,t}$	cm^2	D squared [25]	
Shade	$S_{ij,t}$	ordinal	1 - 5 FIA/NEON classes	MASTIF
Moisture deficit M	M_j	cm	$\sum_{m=1}^{12}(PET_{j,m,t} - P_{j,m,t})$	terraClimate, CHELSA
M anomaly	$M_{j,t}$	cm	anomaly for site	terraClimate, CHELSA
Temperature	T_j	°C	site T	terraClimate, CHELSA
	T_j^2	°C^2	site T squared	
T anomaly	$T_{j,t}$	°C	anomaly for site	terraClimate, CHELSA
$M : D$	$M_j D_{ij,t}$	cm × cm	$M : D$ interaction	
Cation exchange capacity (0 - 30cm)	CEC_j	mmolc/kg	site fertility	soilgrid250m [19]
Slope, aspect	$\mathbf{u}_j$	radians	site drainage	SRTM, NED

T and M includes both current year and previous year. Downscaling of climate variable with terraClimate and CHELSA can be found in the main texts. SRTM represents Shuttle Radar Topography Mission from NASA and NED is National Elevation Dataset from USGS

Supplementary Table 4: An additional regression by adding a factor (i.e., order in the taxonomic rank) as well as its interaction with seed mass to the regression in Fig. 2a of the main texts (R^2 =0.227 for 714 species). (a) summarizes the results from analysis of covariance (ANCOVA) test; (b) provides the linear slope between seed mass and species seed production (SSP, g seed per m^2 tree basal area) and the intercept for each order. The p-value is calculated from a two-tailed test.

(a)

	Df	Sum Sq	Mean Sq	F-value	p-value
seed mass	1	241.73	241.73	174.33	< 0.001
Order	13	41.90	3.22	2.32	0.005
seed mass:Order	13	44.78	3.44	2.48	0.003
Residuals	686	951.19	1.39		

(b)

	slope	Std	t-value	p-value	intercept	Std	t-value	p-value
Ericales	0.315	0.195	1.62	0.11	4.26	0.206	20.6	<0.001
Fabales	0.653	0.288	2.27	0.02	3.92	0.261	15.00	<0.001
Fagales	0.126	0.127	0.995	0.32	4.52	0.155	29.2	<0.001
Gentianales	0.776	0.186	4.18	<0.001	4.86	0.299	16.2	<0.001
Lamiales	0.95	0.243	3.91	<0.001	4.59	0.428	10.7	<0.001
Laurales	0.213	0.283	0.752	0.45	3.69	0.215	17.2	<0.001
Magnoliales	-0.0319	0.411	-0.0776	0.93	3.98	0.415	9.57	<0.001
Malpighiales	0.824	0.134	6.15	<0.001	4.49	0.241	18.7	<0.001
Malvales	0.407	0.263	1.55	0.12	3.76	0.476	7.89	<0.001
Myrtales	0.485	0.159	3.04	0.002	4.35	0.335	13.00	<0.001
Pinales	0.163	0.20	0.811	0.42	3.61	0.370	9.76	<0.001
Rosales	0.713	0.168	4.24	<0.001	4.80	0.295	16.2	<0.001
Sapindales	0.75	0.206	3.64	<0.001	4.60	0.245	18.8	<0.001
Others	0.67	0.0926	7.23	<0.001	4.44	0.159	27.9	<0.001

Supplementary Table 5: Effects of wood density and other factors from main texts on species seed production (SSP, g seed per m^2 tree basal area) in a phylogenetic regression (adjusted R^2 =0.22). Wood density did not have a significant effect on SSP and was thus not analyzed further. The p-value were calculated from a two-tailed test

	Estimate	StdErr	t.value	p.value
Intercept	13.0	11.1	1.18	0.24
Wood Density	-1.55	1.10	-1.41	0.16
Foliar N	-1.22	0.504	-2.43	0.02
Foliar P	-35.4	9.13	-3.88	<0.001
Foliar N $\times$ Foliar P	8.38	3.31	2.53	0.01
Broadleaf deciduous	0.871	1.71	0.510	0.61
Broadleaf evergreen	-0.247	1.70	-0.145	0.88
Scalelike evergreen	1.68	13.4	0.125	0.90

Additional notes

- **supplementary data**: Sample sizes by genus and species.

References

[1] Aaron B. Berdanier and James S. Clark. Divergent reproductive allocation trade-offs with canopy exposure across tree species in temperate forests. *Ecosphere*, 7(6):e01313–n/a, 2016. ISSN 2150-8925. doi: 10.1002/ecs2.1313. URL `http://dx.doi.org/10.1002/ecs2.1313`.

[2] Michał Bogdziewicz, Dave Kelly, Peter A. Thomas, Jonathan G. A. Lageard, and Andrew Hacket-Pain. Climate warming disrupts mast seeding and its fitness benefits in european beech. *Nature Plants*, 6(2):88–94, 2020. ISSN 2055-0278. doi: 10.1038/s41477-020-0592-8. URL `https://doi.org/10.1038/s41477-020-0592-8https://www.nature.com/articles/s41477-020-0592-8.pdf`.

[3] Norman A. Bourg, William J. McShea, Jonathan R. Thompson, Jennifer C. McGarvey, and Xiaoli Shen. Initial census, woody seedling, seed rain, and stand structure data for the scbi sigeo large forest dynamics plot. *Ecology*, 94:2111–2112, 2013. doi: doi.org/10.1890/13-0010.1. URL `https://doi.org/10.1890/13-0010.1`.

[4] Thomas Caignard, Antoine Kremer, Cyril Firmat, Manuel Nicolas, Samuel Venner, and Sylvain Delzon. Increasing spring temperatures favor oak seed production in temperate areas. *Scientific Reports*, 7(1):8555, 2017. ISSN 2045-2322. doi: 10.1038/s41598-017-09172-7. URL `https://doi.org/10.1038/s41598-017-09172-7`.

[5] Xiongwen Chen, Dale G. Brockway, and Qinfeng Guo. Characterizing the dynamics of cone production for longleaf pine forests in the southeastern united states. *Forest Ecology and Management*, 429:1–6, 2018. ISSN 0378-1127. doi: https://doi.org/10.1016/j.foreco.2018.06.014. URL `http://www.sciencedirect.com/science/article/pii/S037811271830553X`.

[6] J. S. Clark, S. LaDeau, and I. Ibanez. Fecundity of trees and the colonization-competition hypothesis. *Ecological Monographs*, 74(3):415–442, 2004. doi: 10.1890/02-4093.

[7] J. S. Clark, D. M. Bell, M. C. Kwit, and K. Zhu. Competition-interaction landscapes for the joint response of forests to climate change. *Global Change Biology*, 20(6):1979–1991, 2014. doi: 10.1111/gcb.12425.

[8] Natalie L. Cleavitt and Timothy J. Fahey. Seed production of sugar maple and american beech in northern hardwood forests, new hampshire, usa. *Canadian Journal of Forest Research*, 47(7):985–990, 2017. ISSN 0045-5067. doi: 10.1139/cjfr-2017-0096. URL `https://doi.org/10.1139/cjfr-2017-0096`.

[9] Evangelia N. Daskalakou, Katerina Koutsovoulou, Kostas Ioannidis, Panagiotis P. Koulelis, Petros Ganatsas, and Costas A. Thanos. Masting and regeneration dynamics of abies cephalonica, the greek endemic silver fir. *Seed Science Research*, 29(4):227–237, 2019. ISSN 0960-2585. doi: 10.1017/S0960258519000205. URL `https://www.cambridge.org/core/article/masting-and-regeneration-dynamics-of-abies-cephalonica-the-greek-endemic-silver-fir/A27C2258BF34F905AC62B5F8B2C29370`.

[10] Hendrik Davi, Maxime Cailleret, Gwendal Restoux, Annabelle Amm, Christian Pichot, and Bruno Fady. Disentangling the factors driving tree reproduction. *Ecosphere*, 7(9):e01389, 2016. ISSN 2150-8925. doi: https://doi.org/10.1002/ecs2.1389. URL `https://esajournals.onlinelibrary.wiley.com/doi/abs/10.1002/ecs2.1389`.

[11] Ester González de Andrés, J. Julio Camarero, Isabel Martínez, and Lluis Coll. Uncoupled spatiotemporal patterns of seed dispersal and regeneration in pyrenean silver fir populations. *Forest Ecology and Management*, 319:18–28, 2014. ISSN 0378-1127. doi: https://doi.org/10.1016/j.foreco.2014.01.050. URL http://www.sciencedirect.com/science/article/pii/S0378112714000747.

[12] S. Debain, T. Curt, J. Lepart, and B. Prevosto. Reproductive variability in pinus sylvestris in southern france: Implications for invasion. *Journal of Vegetation Science*, 14(4):509–516, 2003. ISSN 1100-9233. doi: https://doi.org/10.1111/j.1654-1103.2003.tb02177.x. URL https://doi.org/10.1111/j.1654-1103.2003.tb02177.x.

[13] Laurent Dormont, Werner Baltensweiler, Rémi Choquet, and Alain Roques. Larch- and pine-feeding host races of the larch bud moth (zeiraphera diniana) have cyclic and synchronous population fluctuations. *Oikos*, 115(2):299–307, 2006. ISSN 0030-1299. doi: https://doi.org/10.1111/j.2006.0030-1299.15010.x. URL https://onlinelibrary.wiley.com/doi/abs/10.1111/j.2006.0030-1299.15010.x.

[14] Violette Doublet, Cindy Gidoin, François Lefèvre, and Thomas Boivin. Spatial and temporal patterns of a pulsed resource dynamically drive the distribution of specialist herbivores. *Scientific Reports*, 9(1):17787, 2019. ISSN 2045-2322. doi: 10.1038/s41598-019-54297-6. URL https://doi.org/10.1038/s41598-019-54297-6.

[15] Andrew Hacket-Pain, Davide Ascoli, Roberta Berretti, Maurizio Mencuccini, Renzo Motta, Paola Nola, Pietro Piussi, Flavio Ruffinatto, and Giorgio Vacchiano. Temperature and masting control norway spruce growth, but with high individual tree variability. *Forest Ecology and Management*, 438:142–150, 2019. ISSN 0378-1127. doi: https://doi.org/10.1016/j.foreco.2019.02.014. URL http://www.sciencedirect.com/science/article/pii/S0378112718320139.

[16] Arndt Hampe and Franz Bairlein. Modified dispersal-related traits in disjunct populations of bird-dispersed frangula alnus (rhamnaceae): a result of its quaternary distribution shifts? *Ecography*, 23(5):603–613, 2000. ISSN 0906-7590. doi: https://doi.org/10.1111/j.1600-0587.2000.tb00179.x. URL https://onlinelibrary.wiley.com/doi/abs/10.1111/j.1600-0587.2000.tb00179.x.

[17] Qingmin Han, Daisuke Kabeya, Atsuhiro Iio, Yoshiyuki Inagaki, and Yoshitaka Kakubari. Nitrogen storage dynamics are affected by masting events in faguscrenata. *Oecologia*, 174(3):679–687, 2014. doi: 10.1007/s00442-013-2824-3.

[18] Mick E Hanley, Benjamin I Cook, and Michael Fenner. Climate variation, reproductive frequency and acorn yield in english oaks. *Journal of Plant Ecology*, 12(3):542–549, 2018. ISSN 1752-993X. doi: 10.1093/jpe/rty046. URL https://doi.org/10.1093/jpe/rty046.

[19] Tomislav Hengl, Jorge Mendes de Jesus, Gerard B. M. Heuvelink, Maria Ruiperez Gonzalez, Milan Kilibarda, Aleksandar Blagotić, Wei Shangguan, Marvin N. Wright, Xiaoyuan Geng, Bernhard Bauer-Marschallinger, Mario Antonio Guevara, Rodrigo Vargas, Robert A. MacMillan, Niels H. Batjes, Johan G. B. Leenaars, Eloi Ribeiro, Ichsani Wheeler, Stephan Mantel, and Bas Kempen. Soilgrids250m: Global gridded soil information based on machine learning. *Plos One*, 12(2):1–40, 02 2017. doi: 10.1371/journal.pone.0169748.

[20] Johannes M. H. Knops and Walter D. Koenig. Sex allocation in california oaks: Trade-offs or resource tracking? *PLOS ONE*, 7(8):e43492, 2012. doi: 10.1371/journal.pone.0043492. URL `https://doi.org/10.1371/journal.pone.0043492`.

[21] Yassine Messaoud, Yves Bergeron, and Hugo Asselin. Reproductive potential of balsam fir (abies balsamea), white spruce (picea glauca), and black spruce (p. mariana) at the ecotone between mixedwood and coniferous forests in the boreal zone of western quebec. *American Journal of Botany*, 94(5):746–754, 2007. ISSN 0002-9122. doi: 10.3732/ajb.94.5.746. URL `https://bsapubs.onlinelibrary.wiley.com/doi/abs/10.3732/ajb.94.5.746`.

[22] Julian M. Norghauer and David M. Newbery. Tree size and fecundity influence ballistic seed dispersal of two dominant mast-fruiting species in a tropical rain forest. *Forest Ecology and Management*, 338:100–113, 2015. ISSN 0378-1127. doi: https://doi.org/10.1016/j.foreco.2014.11.005. URL `https://www.sciencedirect.com/science/article/pii/S0378112714006616`.

[23] Robert R. Parmenter, Roman I. Zlotin, Douglas I. Moore, and Orrin B. Myers. Environmental and endogenous drivers of tree mast production and synchrony in piñon–juniper–oak woodlands of new mexico. *Ecosphere*, 9(8):e02360, 2018. ISSN 2150-8925. doi: 10.1002/ecs2.2360. URL `https://esajournals.onlinelibrary.wiley.com/doi/abs/10.1002/ecs2.2360`.

[24] Ignacio M. Pérez-Ramos, Cristina Aponte, Luis V. García, Carmen M. Padilla-Díaz, and Teodoro Marañón. Why is seed production so variable among individuals? a ten-year study with oaks reveals the importance of soil environment. *PLOS ONE*, 9(12):e115371, 2014. doi: 10.1371/journal.pone.0115371. URL `https://doi.org/10.1371/journal.pone.0115371`.

[25] Tong Qiu, Marie-Claire Aravena, Robert Andrus, Davide Ascoli, Yves Bergeron, Roberta Berretti, Michal Bogdziewicz, Thomas Boivin, Raul Bonal, Thomas Caignard, Rafael Calama, J. Julio Camarero, Connie J. Clark, Benoit Courbaud, Sylvain Delzon, Sergio Donoso Calderon, William Farfan-Rios, Catherine A. Gehring, Gregory S. Gilbert, Cathryn H. Greenberg, Qinfeng Guo, Janneke Hille Ris Lambers, Kazuhiko Hoshizaki, Ines Ibanez, Valentin Journé, Christopher L. Kilner, Richard K. Kobe, Walter D. Koenig, Georges Kunstler, Jalene M. LaMontagne, Mateusz Ledwon, James A. Lutz, Renzo Motta, Jonathan A. Myers, Thomas A. Nagel, Chase L. Nuñez, Ian S. Pearse, Łukasz Piechnik, John R. Poulsen, Renata Poulton-Kamakura, Miranda D. Redmond, Chantal D. Reid, Kyle C. Rodman, C. Lane Scher, Harald Schmidt Van Marle, Barbara Seget, Shubhi Sharma, Miles Silman, Jennifer J. Swenson, Margaret Swift, Maria Uriarte, Giorgio Vacchiano, Thomas T. Veblen, Amy V. Whipple, Thomas G. Whitham, Andreas P. Wion, S. Joseph Wright, Kai Zhu, Jess K. Zimmerman, Magdalena Żywiec, and James S. Clark. Is there tree senescence? the fecundity evidence. *Proceedings of the National Academy of Sciences*, 118(34), 2021. doi: 10.1073/pnas.2106130118.

[26] Miranda D. Redmond, Frank Forcella, and Nichole N. Barger. Declines in pinyon pine cone production associated with regional warming. *Ecosphere*, 3(12):art120, 2012. ISSN 2150-8925. doi: 10.1890/es12-00306.1. URL `https://esajournals.onlinelibrary.wiley.com/doi/abs/10.1890/ES12-00306.1`.

[27] Kyle C. Rodman, Thomas T. Veblen, Teresa B. Chapman, Monica T. Rother, Andreas P. Wion, and Miranda D. Redmond. Limitations to recovery following wildfire in dry forests

of southern colorado and northern new mexico, usa. *Ecological Applications*, 30(1):e02001, 2020. ISSN 1051-0761. doi: 10.1002/eap.2001. URL https://esajournals.onlinelibrary.wiley.com/doi/abs/10.1002/eap.2001.

[28] Anita K. Rose, Cathryn H. Greenberg, and Todd M. Fearer. Acorn production prediction models for five common oak species of the eastern united states. *The Journal of Wildlife Management*, 76(4):750–758, 2012. ISSN 0022-541X. doi: 10.1002/jwmg.291. URL https://wildlife.onlinelibrary.wiley.com/doi/abs/10.1002/jwmg.291.

[29] JAVIER SANGUINETTI and THOMAS KITZBERGER. Patterns and mechanisms of masting in the large-seeded southern hemisphere conifer araucaria araucana. *Austral Ecology*, 33(1):78–87, 2008. ISSN 1442-9985. doi: https://doi.org/10.1111/j.1442-9993.2007.01792.x. URL https://onlinelibrary.wiley.com/doi/abs/10.1111/j.1442-9993.2007.01792.x.

[30] Jacob N. Straub, Richard M. Kaminski, Alan G. Leach, Andrew W. Ezell, and Theodor Leininger. Acorn yield and masting traits of red oaks in the lower mississippi river alluvial valley. *Forest Science*, 62(1):18–27, 2016. ISSN 0015-749X. doi: 10.5849/forsci.14-152. URL https://doi.org/10.5849/forsci.14-152.

[31] M. Uriarte, J. S. Clark, J. K. Zimmerman, L. S. Comita, J. Forero-Montana, and J. Thompson. Multidimensional trade-offs in species responses to disturbance: implications for diversity in a subtropical forest. *Ecology*, 93(1):191–205, 2012. ISSN 0012-9658. URL <GotoISI>://WOS:000301996100021.

[32] Phillip J. van Mantgem, Nathan L. Stephenson, and Jon E. Keeley. Forest reproduction along a climatic gradient in the sierra nevada, california. *Forest Ecology and Management*, 225(1-3):391–399, 2006. doi: 10.1016/j.foreco.2006.01.015. URL http://pubs.er.usgs.gov/publication/70030395.

[33] J.N. Viglas, C.D. Brown, and J.F. Johnstone. Age and size effects on seed productivity of northern black spruce. *Canadian Journal of Forest Research-Revue Canadienne De Recherche Forestiere*, 43:534–543, 2013. doi: doi.org/10.1139/cjfr-2013-0022.

[34] Amy V. Whipple, Neil S. Cobb, Catherine A. Gehring, Susan Mopper, Lluvia Flores-Rentería, and Thomas G. Whitham. Long-term studies reveal differential responses to climate change for trees under soil- or herbivore-related stress. *Frontiers in Plant Science*, 10(132), 2019. ISSN 1664-462X. doi: 10.3389/fpls.2019.00132. URL https://www.frontiersin.org/article/10.3389/fpls.2019.00132.

[35] Andreas P. Wion, Peter J. Weisberg, Ian S. Pearse, and Miranda D. Redmond. Aridity drives spatiotemporal patterns of masting across the latitudinal range of a dryland conifer. *Ecography*, 43(4):569–580, 2020. ISSN 0906-7590. doi: https://doi.org/10.1111/ecog.04856. URL https://onlinelibrary.wiley.com/doi/abs/10.1111/ecog.04856.

[36] Boyd R. Wright and Alain F. Zuur. Seedbank dynamics after masting in mulga (acacia aptaneura): Implications for post-fire regeneration. *Journal of Arid Environments*, 107:10–17, 2014. ISSN 0140-1963. doi: https://doi.org/10.1016/j.jaridenv.2014.03.008. URL https://www.sciencedirect.com/science/article/pii/S0140196314000755.

[37] Micah C. Wright, Phillip van Mantgem, Nathan L. Stephenson, Adrian J. Das, and Jon E. Keeley. Data from: Seed source, not drought, determines patterns of seed production in Sierra Nevada conifers, 2020. URL https://doi.org/10.5066/P9B425MF.

[38] Micah C. Wright, Phillip van Mantgem, Nathan L. Stephenson, Adrian J. Das, and Jon E. Keeley. Seed production patterns of surviving sierra nevada conifers show minimal change following drought. *Forest Ecology and Management*, 480:118598, 2021. ISSN 0378-1127. doi: https://doi.org/10.1016/j.foreco.2020.118598. URL https://www.sciencedirect.com/science/article/pii/S0378112720313670.
