# Supplementary material for: Limits to reproduction and seed size-number trade-offs that shape forest dominance and future recovery
Source: Nat Commun. 2022 May 2;13:2381. doi: 10.1038/s41467-022-30037-9 (PMC9061860; doi:10.1038/s41467-022-30037-9)
Supplement: Supplementary file 2 — Reporting Summary [file 41467_2022_30037_MOESM2_ESM.pdf]

nature portfolio

Corresponding author(s): James S. Clark

Last updated by author(s): Mar 30, 2022

Reporting Summary

Nature Portfolio wishes to improve the reproducibility of the work that we publish. This form provides structure for consistency and transparency in reporting. For further information on Nature Portfolio policies, see our Editorial Policies and the Editorial Policy Checklist.

Statistics

For all statistical analyses, confirm that the following items are present in the figure legend, table legend, main text, or Methods section.

n/a	Confirmed	
☐	☒	The exact sample size (*n*) for each experimental group/condition, given as a discrete number and unit of measurement
☐	☒	A statement on whether measurements were taken from distinct samples or whether the same sample was measured repeatedly
☐	☒	The statistical test(s) used AND whether they are one- or two-sided<br>*Only common tests should be described solely by name; describe more complex techniques in the Methods section.*
☐	☒	A description of all covariates tested
☐	☒	A description of any assumptions or corrections, such as tests of normality and adjustment for multiple comparisons
☐	☒	A full description of the statistical parameters including central tendency (e.g. means) or other basic estimates (e.g. regression coefficient) AND variation (e.g. standard deviation) or associated estimates of uncertainty (e.g. confidence intervals)
☐	☒	For null hypothesis testing, the test statistic (e.g. *F*, *t*, *r*) with confidence intervals, effect sizes, degrees of freedom and *P* value noted<br>*Give P values as exact values whenever suitable.*
☐	☒	For Bayesian analysis, information on the choice of priors and Markov chain Monte Carlo settings
☐	☒	For hierarchical and complex designs, identification of the appropriate level for tests and full reporting of outcomes
☒	☐	Estimates of effect sizes (e.g. Cohen's *d*, Pearson's *r*), indicating how they were calculated

Our web collection on statistics for biologists contains articles on many of the points above.

Software and code

Policy information about availability of computer code

Data collection	No software was used for data collection.
Data analysis	R statistical software (v 4.0.2) was used in this work. All analyses used published R packages, with details stated in the section Methods. The packages included phytools v0.7-70, phylolm v2.6.2, mastif v1.0.1

For manuscripts utilizing custom algorithms or software that are central to the research but not yet described in published literature, software must be made available to editors and reviewers. We strongly encourage code deposition in a community repository (e.g. GitHub). See the Nature Portfolio guidelines for submitting code & software for further information.

Data

Policy information about availability of data

All manuscripts must include a data availability statement. This statement should provide the following information, where applicable:

- Accession codes, unique identifiers, or web links for publicly available datasets
- A description of any restrictions on data availability
- For clinical datasets or third party data, please ensure that the statement adheres to our policy

Seed production data are available at the Duke Data Repository (https://doi.org/10.7924/r4348ph5t). Species traits are downloaded from TRY Plant Trait database at https://www.try-db.org/TryWeb/Home.php. Cation exchange capacity data are obtained at https://soilgrids.org/. Climate data are extracted from Terraclimate at http://www.climatologylab.org/ and CHELSA at https://chelsa-climate.org/. Elevation data are obtained from SRTM at https://srtm.csi.cgiar.org/ and USGS National Elevation Dataset at https://ned.usgs.gov/.

Field-specific reporting

Please select the one below that is the best fit for your research. If you are not sure, read the appropriate sections before making your selection.

☐ Life sciences ☐ Behavioural & social sciences ☒ Ecological, evolutionary & environmental sciences

For a reference copy of the document with all sections, see nature.com/documents/nr-reporting-summary-flat.pdf

Ecological, evolutionary & environmental sciences study design

All studies must disclose on these points even when the disclosure is negative.

Study description	We quantified seed production from 736,759 trees and 12,063,723 tree-years from 751 species in 6 continents that are available to authors. We used two data types including crop counts and seed traps.
Research sample	Each sample is a tree-year observation, i.e., an individual tree at a given year. Sample sizes, sites, and citation of data source is detailed in Table S2. Sample size by each tree species is detailed in the supplementary data file
Sampling strategy	This study use all data from crop counts and seed traps. The methods that accounts for dependences between the two data types are detailed in the paper with citation Clark et al., 2019 (Ecological Monographs).
Data collection	Seed traps were used to capture seeds falling from trees. Seed trap data were collected by emptying the traps multiple times each year. Crop counts were collected with binoculars. All authors collected data.
Timing and spatial scale	Observations ranged from year 1960 to the present and were collected in North America, South and Central America, Africa, Europe, Asia, and Oceania. Seed trap were emptied multiple times every year. Crop counts were collected annually when trees were fruiting. Fig S1 contains a map of sites. Table S2 contains sample size by sites.
Data exclusions	No data were excluded in this study.
Reproducibility	No experiments were performed in this study. Previous published data and existing R packages from CRAN were used in this study. Results can be reproduced following the method section.
Randomization	Randomization is not applicable because no experiments were performed in this study
Blinding	Blinding is not applicable because no experiments were performed in this study

Did the study involve field work? ☒ Yes ☐ No

Field work, collection and transport

Field conditions	The climate conditions can be assessed using the coordinates of each plot, which are shown as a map in Fig S1 and are detailed in Table S2
Location	Fig. S1 contains the map of locations and table S2 includes locations.
Access & import/export	Access were determined by individual PI, which is detailed in table S2.
Disturbance	No disturbance was caused by the study

Reporting for specific materials, systems and methods

We require information from authors about some types of materials, experimental systems and methods used in many studies. Here, indicate whether each material, system or method listed is relevant to your study. If you are not sure if a list item applies to your research, read the appropriate section before selecting a response.

Materials & experimental systems

n/a	Involved in the study	
☒	☐	Antibodies
☒	☐	Eukaryotic cell lines
☒	☐	Palaeontology and archaeology
☒	☐	Animals and other organisms
☒	☐	Human research participants
☒	☐	Clinical data
☒	☐	Dual use research of concern

Methods

n/a	Involved in the study	
☒	☐	ChIP-seq
☒	☐	Flow cytometry
☒	☐	MRI-based neuroimaging
