# Supplementary material for: Limits to reproduction and seed size-number trade-offs that shape forest dominance and future recovery
Source: Nat Commun. 2022 May 2;13:2381. doi: 10.1038/s41467-022-30037-9 (PMC9061860; doi:10.1038/s41467-022-30037-9)
Supplement: Supplementary file 4 — Description of Additional Supplementary Files [file 41467_2022_30037_MOESM4_ESM.pdf]

Title: Supplementary data 1:
Description: sample size by genus and species
